# Cigarette smoking and risk of acoustic neuromas and pituitary tumours in the Million Women Study

**DOI:** 10.1038/sj.bjc.6605695

**Published:** 2010-05-11

**Authors:** V S Benson, J Green, K Pirie, V Beral

**Affiliations:** 1 Cancer Epidemiology Unit, Nuffield Department of Clinical Medicine, University of Oxford, Richard Doll Building, Roosevelt Drive, Oxford OX3 7LF, UK

**Keywords:** acoustic neuroma, pituitary tumour, smoking, women

## Abstract

**Background:**

The relationship between cigarette smoking and incidence of acoustic neuromas and pituitary tumours is uncertain.

**Methods:**

We examined the relation between smoking and risk of acoustic neuromas and pituitary tumours in a prospective study of 1.2 million middle-aged women in the United Kingdom.

**Results:**

Over 10.2 million person years of follow-up, 177 women were diagnosed with acoustic neuromas and 174 with pituitary tumours. Current smokers at recruitment were at significantly reduced risk of incident acoustic neuroma compared with never smokers (adjusted relative risk (RR)=0.41, 95% confidence interval (CI)=0.24–0.70, *P*=0.001). Past smokers did not have significantly different risk of acoustic neuroma than never smokers (RR=0.87, 95% CI=0.62–1.22, *P*=0.4). Smoking was not associated with incidence of pituitary tumours (RR in current *vs* never smokers=0.91, 95% CI=0.60–1.40, *P*=0.7).

**Conclusion:**

Women who smoke are at a significantly reduced risk of acoustic neuromas, but not of pituitary tumours, compared with never smokers. Acoustic neuromas are much rarer than the cancers that are increased among smokers.

Acoustic neuromas and pituitary tumours are rare, each accounting for approximately 10% of all intracranial tumours. Acoustic neuromas are slow-growing benign tumours arising from Schwann cells of the eighth cranial nerve (Propp *et al*, 2006). Pituitary tumours are typically benign adenomas and may be hormone secreting or non-functioning (Asa and Ezzat, 2009). The aetiology of both tumour types is unclear (Evans *et al*, 2005; McGregor, 2009), with no firmly established common environmental risk factors. However, recent results from a multicentre case–control study showed a significantly lower risk for acoustic neuroma in current smokers than in never smokers (Schoemaker *et al*, 2007); no association was found between smoking and incidence of pituitary tumour (Schoemaker and Swerdlow, 2009).

We examined the relation between smoking and incident acoustic neuromas and pituitary tumours in a large prospective study of middle-aged women in the United Kingdom.

## Materials and methods

During 1996–2001, 1.3 million middle-aged women (mean age 56 years) were recruited into the Million Women Study cohort, completing a recruitment questionnaire about socio-demographic factors, reproductive and medical history and other personal characteristics, including details on smoking status. Full details of the study design and methods are described elsewhere (Million Women Study Collaborative Group, 1999) and the questionnaire can be viewed at http://www.millionwomenstudy.org.

All study participants have been flagged on the National Health Service (NHS) Central Registers and tumour registrations and deaths are routinely notified to the study investigators. This information includes the date of each such event and codes the tumour site and morphology using the 10th revision of the International Classification of Diseases (ICD-10) (World Health Organization, 1992). Follow-up is complete for over 99% of the study population.

Acoustic neuromas were defined as those coded as ICD-10 D33.3, with morphology code ICD-O 9650/0. Pituitary tumours were defined as those coded as ICD-10 C75.1, D35.2 or D44.3.

Women were classed as current, past or never smokers, as reported at recruitment. Current smokers were further classified according to the average number (<15, 15+) of cigarettes smoked per day.

### Statistical analysis

Women were excluded from the analyses if at recruitment they had been diagnosed with any type of invasive tumour (other than non-melanoma skin cancer [C44]) or any non-invasive tumour of the CNS, or if there was no information on smoking status. Women were also excluded if they reported having the inherited disorder neurofibromatosis (Q85.0) at recruitment.

Eligible women contributed person years from the date of recruitment until the date of registration of an acoustic neuroma or a pituitary tumour, date of death or end of follow-up, whichever was the earliest. In addition, women diagnosed with any cancer (except non-melanoma skin cancer) or any non-invasive CNS tumour during the follow-up period were censored at the date of diagnosis of that tumour, to avoid potential biases because of the effect of treatment or of altered surveillance. The end of follow-up for tumour incidence was 31 December 2007 for East Anglia, South West and North West (Mersey) regions, 30 June 2007 for Oxford, Thames, West Midlands and Trent and 31 December 2006 for North Yorkshire, North West (Manchester/Lancashire) and Scotland.

Relative risks (RRs) and 95% confidence intervals (CIs) were obtained using Cox proportional hazards models with attained age as the underlying time variable. The proportional hazards assumption was assessed using tests based on Schoenfeld residuals, which showed no evidence of a violation for any of the exposure comparisons listed in the tables.

Analyses were stratified by region and by age at recruitment, and as there are no established common environmental risk factors for either tumour site, we examined the effect of adjusting for the following potential risk factors separately, and for all simultaneously: socio-economic status (quintiles based on Townsend deprivation index; Townsend *et al*, 1988), height (<160, 160–164.9, ⩾165 cm), body mass index (<25, 25–29.9, ⩾30 kg m^–2^), alcohol intake (never, <7, ⩾7 drinks per week), strenuous exercise (<1, 1, ⩾2 times per week), parity (nulliparous, 1–2 and 3+ full-term pregnancies), age at first birth (<20, 20–24, 25+ years), menopausal status (pre/perimenopausal, <5 years postmenopause, 5+ years postmenopause) and use of oral contraceptives (never, <5, ⩾5 years of use) and of hormone therapy for the menopause (never, past, current). Women with missing values for any of the adjustment variables were assigned to a separate category for that variable.

## Results

In total, 1 240 593 women aged 56 years on average at recruitment were included in the analyses. During 10.2 million person years of follow-up (an average of 8.2 years per woman), 177 acoustic neuromas and 174 pituitary tumours were registered. Table 1 shows characteristics of the study population by smoking status. A total of 607 215 (49%) women had ever smoked, and 254 992 (21%) were current smokers.

Women who had ever smoked had a significantly decreased risk of acoustic neuroma compared with never smokers (adjusted RR=0.69, 95% CI=0.51–0.95, *P*=0.02). As shown in Table 2, risk was significantly lower in current smokers (RR compared with never smokers=0.41, 95% CI=0.24–0.70, *P*=0.001) than in past smokers (RR compared with never smokers=0.87, 95% CI=0.62–1.22, *P*=0.4): *P* for heterogeneity=0.006. Although the reduction in risk in current smokers seemed to be greater with increasing numbers of cigarettes smoked (RRs=0.53 *vs* 0.27 in women who smoked <15 and 15+ cigarettes per day, respectively), this difference was not statistically significant (*P* for heterogeneity=0.2). Smoking was not associated with incidence of pituitary tumours (RR for ever *vs* never smokers=0.99, 95% CI=0.73–1.35, *P*=0.95; for current *vs* never smokers, 0.91, 95% CI=0.60–1.40, *P*=0.7). No material effect on the RRs was seen with either individual or simultaneous (Table 2) adjustment for the 10 potential confounding factors considered.

## Discussion

In this large prospective study, incidence of acoustic neuroma was significantly and substantially decreased in current smokers. Incidence of pituitary tumours was not significantly associated with smoking. Our findings are consistent with the results from the only relevant earlier epidemiological study, a case–control study including 563 acoustic neuromas (RR for current *vs* never smokers=0.5; Schoemaker *et al*, 2007) and 299 pituitary tumours (RR for ever *vs* never smokers=1.2; Schoemaker and Swerdlow, 2009).

Strengths of this investigation include the prospective study design, large study size and complete and non-differential follow-up for cancer incidence. Despite the large sample size, the numbers of cases were still relatively small and the study had limited power to investigate smoking by amount or duration.

No clear association has been found between smoking and incidence of other tumours of the CNS, including glioma or meningioma, either in this cohort (Benson *et al*, 2008) or in other studies (Mandelzweig *et al*, 2009). Possible mechanisms for an association between smoking and risk of acoustic neuroma include, as well as direct effects of tobacco carcinogens, the effects of cigarette smoking on hormonal status (Kapoor and Jones, 2005); female sex hormones may have a function in development of some central nervous system tumours, including acoustic neuroma (Benson *et al*, 2010). A reduced risk of acoustic neuromas among smokers, if confirmed, would have minimal implications for public health: acoustic neuromas are much rarer than the cancers that are increased among smokers.

## Figures and Tables

**Table 1:** Characteristics of the study population, by smoking status at recruitment

	**Current smoker (*n*=254 992)**	**Past smoker (*n*=352 223)**	**Never smoked (*n*=633 378)**
*Characteristics at recruitment*			
Mean age, years (s.d.)	55.3 (4.5)	56.3 (4.9)	56.3 (4.9)
Socio-economic group (% in upper third)	23	33	38
Past use of oral contraceptives (%)	65	64	56
Mean parity (s.d.)	2.3	2.1	2.1
Mean height, cm (s.d.)	161.5 (6.8)	162.3 (6.7)	162.0 (6.7)
Mean body mass index, kg m^–2^ (s.d.)	25.6 (4.5)	26.7 (4.8)	26.2 (4.6)
Strenuous physical activity ⩾once per week (%)	30	41	41
Mean alcohol intake, g per week (s.d.)	47.1 (59.6)	53.8 (58.7)	37.1 (45.8)
Current use of hormone replacement therapy (%)	36	36	31
			
*Follow-up for incident acoustic neuromas and pituitary tumours*			
Person-years of follow-up (millions)	2.05	2.88	5.23
Number of incident acoustic neuroma cases	16	52	109
Number of incident pituitary tumour cases	31	52	91

**Table 2:** Relative risks (RRs) and 95% confidence intervals (CIs) for incident acoustic neuroma and pituitary tumour in relation to smoking status in the Million Women Study cohort

	**Acoustic neuroma**			**Pituitary tumours**		
**Smoking status**	***n* cases**	**RR^a^ (95%CI)**	**RR^b^ (95% CI)**	***n* cases**	**RR^a^ (95%CI)**	**RR^b^ (95% CI)**
Never smoker	109	1.00	1.00	91	1.00	1.00
Past smoker	52	0.87 (0.62–1.21)	0.87 (0.62–1.22)	52	1.04 (0.74–1.47)	1.04 (0.74–1.47)
						
*Current smoker*	16	0.38 (0.22–0.64)	0.41 (0.24–0.70)	31	0.88 (0.59–1.33)	0.91 (0.60–1.40)
Current <15 cigarettes/day	11	0.50 (0.27–0.93)	0.53 (0.28–1.00)	21	1.14 (0.71–1.84)	1.19 (0.73–1.94)
Current 15+ cigarettes/day	5	0.25 (0.10–0.61)	0.27 (0.11–0.66)	10	0.60 (0.31–1.15)	0.60 (0.31–1.18)

a RRs stratified by region and by age at recruitment, with attained age as the underlying time variable.

b RRs stratified by region and by age at recruitment, and adjusted for socio-economic status, alcohol intake, height, body mass index, strenuous exercise, parity, age at first birth, menopausal status and use of oral contraceptives or hormone therapy for the menopause, with attained age as the underlying time variable.
